# Characterization of the dynamics of *Plasmodium falciparum* deoxynucleotide-triphosphate pool in a stage-specific manner

**DOI:** 10.1038/s41598-022-23807-4

**Published:** 2022-11-19

**Authors:** Réka Babai, Richard Izrael, Beáta G. Vértessy

**Affiliations:** 1 Malaria Research Laboratory, Institute of Enzymology, Research Centre for Natural Sciences, Budapest, 1117 Hungary; 2 George A. Olah Doctoral School of Chemistry and Chemical Technology, BME Budapest University of Technology and Economics, Budapest, 1111 Hungary; 3 Department of Applied Biotechnology and Food Sciences, BME Budapest University of Technology and Economics, Budapest, 1111 Hungary; 4 Doctoral School of Multidisciplinary Medical Sciences, University of Szeged, Szeged, 6720 Hungary

**Keywords:** DNA, Enzymes, Metabolomics, DNA metabolism, DNA replication, Transcriptomics, Parasitology, Pathogens

## Abstract

Understanding and characterizing the molecular background of the maintenance of genomic integrity might be a major factor in comprehending the exceptional ability of the malaria parasite, *Plasmodium falciparum* to adapt at a fast pace to antimalarials. A balanced nucleotide pool is an essential factor for high-fidelity replication. The lack of detailed studies on deoxynucleotide-triphosphate (dNTP) pools in various intraerythrocytic stages of *Plasmodium falciparum* motivated our present study. Here, we focused on the building blocks of DNA and utilized an EvaGreen-based dNTP incorporation assay to successfully measure the temporal dynamics of dNTPs in every intraerythrocytic stage and in drug-treated trophozoites. Our findings show that the ratio of dNTPs in the ring-stage parasites significantly differs from the more mature trophozoite and schizont stages. We were also able to detect dGTP levels that have never been shown before and found it to be the least abundant dNTP in all stages. Treatment with WR99210, a TS-DHFR inhibitor drug, affected not only dTTP, but also dGTP levels, despite its presumed selective action on pyrimidine biosynthesis. Results from our studies might assist in a better understanding of genome integrity mechanisms and may potentially lead to novel drug related aspects involving purine and pyrimidine metabolic targets.

## Introduction

Malarial infections threaten hundreds of millions of people yearly, causing severe illness and even death. Despite the effort to eradicate the disease in the last decades, the progress in controlling malaria has slowed down^1^. One of the main underlying reasons is the quick adaptability of the parasite against different antimalarial drugs^2^. Unbalanced dNTP pools potentially lead to misincorporations of deoxynucleotide-triphosphates (dNTPs) during DNA replication giving rise to mutations that promote adaptability to certain antimalarial drugs.

The genome composition of *Plasmodium falciparum* is unique amongst the five human malaria parasites with its exceptionally high AT content. Possessing ~ 80% AT in coding regions and nearly 90% in non-coding regions, *P. falciparum* is not only peculiar amidst the human infecting malaria species but amongst other eukaryotes as well^3^. This phenomenon may contribute to the uniquely superb ability to adapt to new antimalarial drugs. It has been previously published that the GC-to-AT base-pair substitution is overrepresented in the mutational profile of several *Plasmodium falciparum* strains, despite having a working DNA repair machinery^3^. The maintenance of genomic integrity heavily relies on the ratio of deoxynucleotide triphosphates (dNTPs)^4^. A perturbed dNTP pool leads to mismatches during the DNA replication, as the high processivity of DNA polymerase can overcome the lack of complementarity^5^. Furthermore, the low complexity and long stretches of homopolymer DNA tracts can lead to polymerase slippage in the AT-rich sequences, leading to frame-shift mutations, copy number variation and common indels^6^. As parasites naturally grow in an oxidative environment and DNA synthesis proceeds under such circumstances, oxidized bases such as uracil and 8-oxo-guanine might contribute to the biased GC-to-AT transition. We previously demonstrated in a study published in 2018^7^ that the parasites possess significantly elevated levels of uracil, where we utilized a specific uracil DNA sensor developed by our group^8,9^. Uracil may arise from cytidine deamination or thymine-replacing incorporation during DNA replication^10^. The presence of modified, non-canonical bases puts additional pressure on the DNA repair of the parasite to maintain its genomic integrity. The so-called short patch base excision repair, which is more common in eukaryotic organisms during replication due to it requiring less energy to correct the mismatches, is absent in the parasite. The reason behind this is the lack of key DNA polymerase β, thus relying on the repair via the long patch base excision repair. This pathway is more reliant on the availability of nucleotides and requires the repair of longer DNA sequences, thus potentially increasing the chance of mismatches and polymerase slippage^11^.

**Figure 1:**
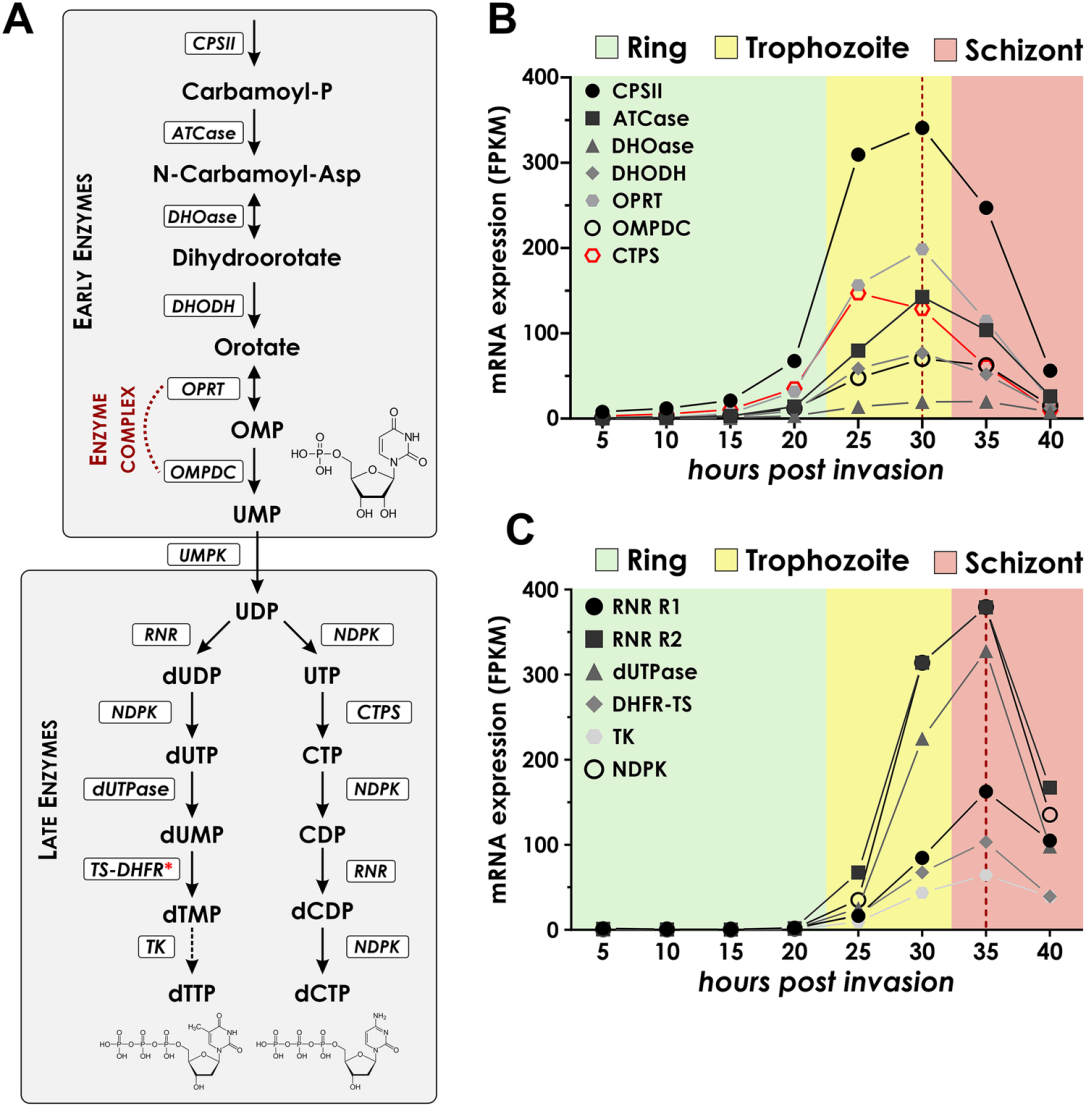
The pyrimidine metabolic pathway of *Plasmodium falciparum*. (**A**) The pathway is initiated by carbamoyl-phosphate synthase II (CPSII) that generates carbamoyl-phosphate. Carbamoylphosphate is turned into an *N*-carbamoyl-L-aspartate by aspartate-transcarbamoylase (ATCase). *N*-carbamoyl-phosphate is cyclized by dihydroorotase (DHOase) to generate dihydroorotate, that is oxidized by the mitochondrial electron transport chain protein dihydroorotate dehydrogenase (DHODH). Finally, an enzyme complex of orotate phosphoribosyl transferase (OPRT) and orotate-5′-monophosphate decarboxylase (OMPDC) turns orotate into orotate-5′-monophosphate (OMP) and immediately decarboxylates it into uridine-5′-monoposphate (UMP). This concludes the early stages of the pyrimidine metabolism, where it branches into the separate thymidine and cytidine biosynthetic processes. For thymidine, UMP is phosphorylated to UDP, then UDP is oxidized by ribonucleotide reductase (RNR) to generate deoxy-UDP (dUDP). dUDP is phosphorylated to dUTP and then it is dephosphorylated to dUMP by the enzyme dUTPase, an essential enzyme to maintain low dUTP levels. Finally, dUMP is turned into thymidine-5′-phosphate (dTMP) by the bifunctional thymidylate synthase-dihydrofolate reductase (TS-DHFR), that is then turned into dTTP in consecutive phosphorylation steps by thymidylate kinase (TK) and nucleotide diphosphate kinase (NDPK). For the cytidine biosynthesis, the sole pathway is through the amination of UTP to cytidine-5′-triphosphate (CTP) by CTP synthase (CTPS). The CTP first must be dephosphorylated into CDP by the nucleoside diphosphate kinase (NDPK) to be turned into deoxy-CDP (dCDP) by the RNR enzyme and phosphorylated back to dCTP consecutively. Dashed lines indicate consecutive phosphorylation steps. Red asterisk marks the enzyme target of WR99210 in the pathway. **(B**,**C**) Transcriptomic analysis of the early (**B**) and late (**C**) enzymes in the pyrimidine metabolic pathways. Transcriptomic data has been gathered from the database generated by *Toenhake* et al.^26^ and reorganized in an orderly manner. Values are shown in fragments per kilobase of exon per million mapped fragments (FPKM) as a measure of expression levels. Green, yellow and red background indicates the stages which we considered to be ring, trophozoite and schizont stages in this study, respectively. The red dashed vertical line shows the peak expression of early and late-stage enzymes, except for CTP synthase (CTPS) that has an earlier peak at 25 h post-invasion, highlighted by the red plot.

The purine and pyrimidine metabolic pathways are conceptually different in parasites as the former is solely through salvage pathway, while pyrimidine synthesis depends completely on de novo synthesis of pyrimidine nucleotides (Fig. 1A)^12^. Although the pyrimidine synthetic pathway is well conserved, the amino acid sequences of the parasitic enzymes involved in the pathway have several notable divergences from their human host^13^. This raises the possibility to develop such antimalarials that act against these crucial enzymes in a species-specific manner, treating only the parasite. The first three steps of pyrimidine synthesis in *Plasmodium falciparum* are the following: (1) formation of carbamoyl phosphate is catalyzed by carbamoyl phosphate synthetase II (CPSII), (2) aspartate-transcarbamoylase (ATCase) places an aspartate to the aforementioned carbamoyl phosphate, (3) the third enzyme of the pathway, dihydroorotase (DHOase) catalyzes the transformation of *N*-carbamoyl-l-aspartate to l-dihydroorotate (DHO). In humans, these steps are catalyzed by a trifunctional protein, called CAD^14,15^. The fourth step is the oxidation of DHO to orotate by dihydroorotate dehydrogenase (DHODH), while an electron pair is transferred to coenzyme Q, making it an attractive drug target as it is tied to the mitochondrial electron transfer system^12,15^. Orotate phosphoribosyltransferase (OPRT) catalyzes the formation of orotidine-5′-monophosphate (OMP) as the fifth step of pyrimidine synthesis. Finally, orotidine-5′-monophosphate decarboxylase (ODC) catalyzes the decarboxylation of OMP to uridine-5′-monophosphate (UMP), the precursor needed for the synthesis of all pyrimidine nucleotides^12,14,15^. In humans, OPRT and ODC are part of a bifunctional enzyme, called UMP synthase^15,16^. The only known way for de novo synthesis of cytidine triphosphate (CTP) in *Plasmodium falciparum* is through CTP synthase (CTPS), which converts uridine triphosphate (UTP) to CTP which through several consecutive steps converts to dCTP. Double phosphorylation of UMP to UTP precedes this process and the converted CTP has to be dephosphorylated to CDP, as the ribonucleotide reductase (RNR) enzyme only oxidizes the nucleotide diphosphates (NDPs) to turn them into deoxynucleotide diphosphates (dNDPs). Deoxythymidine-monophosphate (dTMP) is produced from dUMP via methylation by the bifunctional thymidylate synthase-dihydrofolate reductase (TS-DHFR) enzyme. These corresponding reactions in humans are catalyzed by two separate enzymes^15^. The folate pathway is linked to the pyrimidine synthetic pathway as the oxidation of 5,10-methylenetetrahydrofolate to dihydrofolate is required for dTMP generation. Although mammalian cells are not capable of de novo synthesis of folate cofactors and are acquired via dietary intake, the parasite is capable of de novo folate synthesis as well as salvaging these cofactors^14,17,18^. Several antimalarials act against TS-DHFR such as pyrimethamine and sulfadoxine, which were widely used in clinical practice until the emergence of resistance^19^. Finally, thymidylate kinase (TK) and nucleotide diphosphate kinase (NDPK) phosphorylates dTMP to dTDP and dTTP, respectively, to provide the properly phosphorylated form for replication. An interesting trait of *Pf*TK is the fact that it can also phosphorylate dGMP with comparable enzyme kinetic parameters^20,21^.

**Figure 2:**
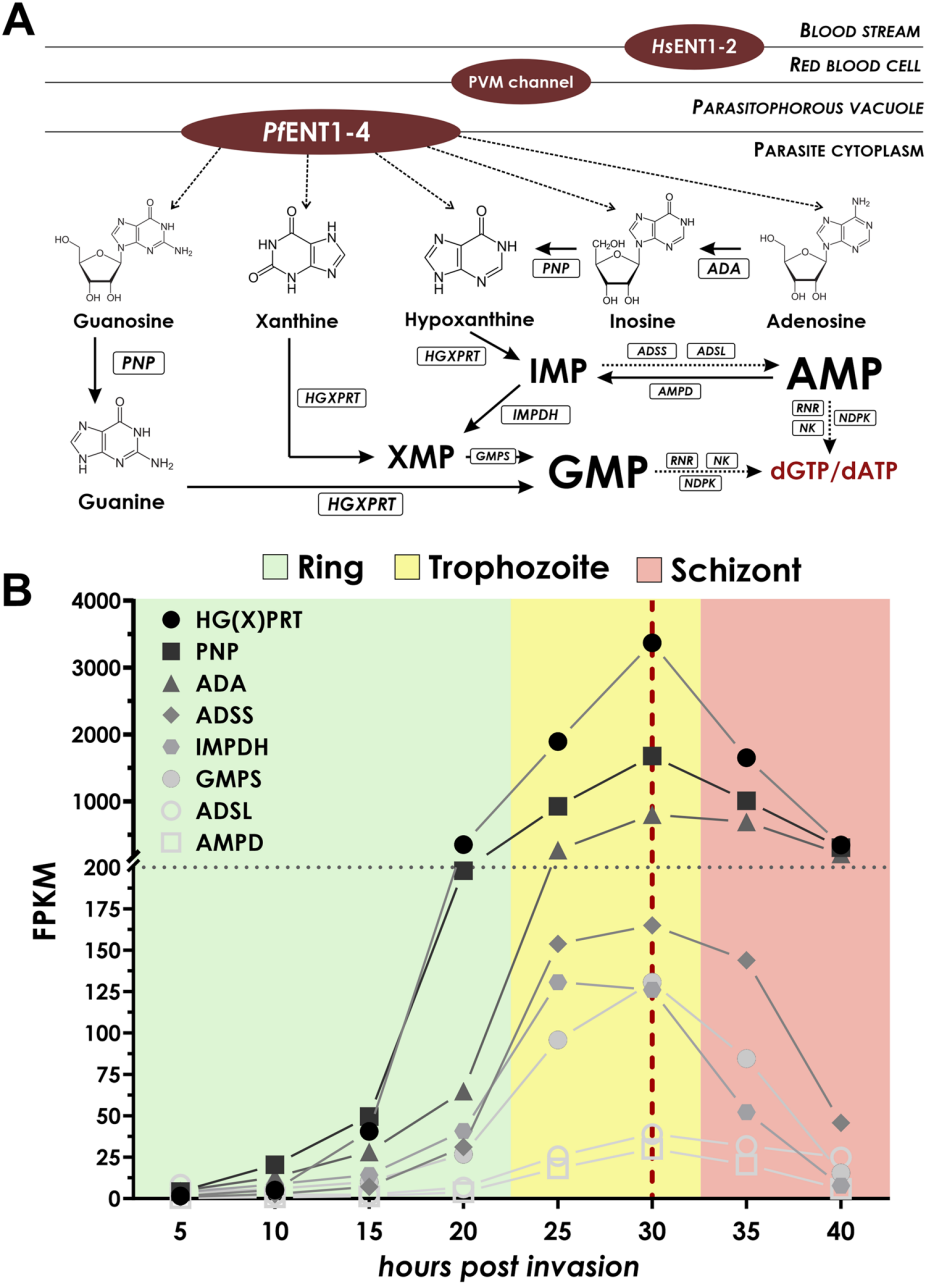
The purine metabolic pathway of *Plasmodium falciparum*. (**A**) The purine metabolic pathway relies on salvaged nucleosides and nucleobases imported from the bloodstream of the host. First, these metabolites are transported through the erythrocyte equilibrative nucleoside transporters (HsENT), which are then passed into the parasitophorous vacuole through the high-throughput parasitophorous vacuole membrane (PVM) channel. The nucleosides are then being transferred into the parasite cytoplasm through the *Pf*ENTs. The nucleosides and nucleobases available are as follows: adenosine, inosine, guanosine, hypoxanthine, xanthine. Firstly, the *N*-glycosidic bonds of nucleosides are cleaved by the purine nucleoside phosphorylase (PNP) enzyme to generate their respective nucleobases. Furthermore, adenosine is first deaminated into inosine by the enzyme adenosine deaminase (ADA). The generated nucleobases are hypoxanthine, xanthine and guanosine that all intertwine in different paths. Most notably, hypoxanthine both can be turned into adenosine- (AMP) and guanosine-5′-monophosphate (GMP). For this, first the hypoxanthine is first transferred onto a phosphoribosyl pyrophosphate (PRPP) by the broad-specific hypoxanthine–guanine-xanthine phosphoribosyl transferase (HGXPRT) to generate inosine-5′-monophosphate (IMP). The IMP can be turned into xanthine-5′-monophosphate (XMP) by the IMP dehydrogenase (IMPDH) enzyme or through two consecutive reactions by adenylosuccinate synthase (ADSS) and lyase (ADSL), it can be converted to AMP. Furthermore, xanthine and guanine can also be transferred onto a PRPP by HGXPRT to generate XMP and guanosine-5′-monophosphate (GMP), respectively. XMP is also used to generate GMP through GMP synthase (GMPS). Finally, in several consecutive steps AMP and GMP can be turned into deoxy-ATP (dATP) and deoxy-GMP (dGMP) after oxidation by ribonucleotide reductase (RNR) and phosphorylation by nucleoside kinase (NK) and nucleoside diphosphate kinase (NDPK). Dashed lines indicate consecutive enzymatic steps. (**B**) Transcriptomic analysis of the enzymes in the purine metabolic pathway. Transcriptomic data has been gathered from the database generated by *Toenhake* et al.^26^. and reorganized in an orderly manner. Values are shown in fragments per kilobase of exon per million mapped fragments (FPKM) as a measure of expression levels. Green, yellow and red background indicates the stages, which we considered to be ring, trophozoite and schizont stages in this study, respectively. The red dashed vertical line shows the peak expression of all purine metabolic enzymes. Note the break on the vertical axis noted by the grey dotted line.

Contrariwise, purine nucleotides are obtained solely through salvage pathway. During the intraerythrocytic stage, the parasites salvage the purine materials from the host erythrocytes (Fig. 2A). Purine nucleobases and nucleosides are obtained by the erythrocytes from plasma through membrane transporters, like equilibrative nucleoside transporters (*Hs*ENT) and facilitated nucleobase transporter (*Hs*FNT) as they also lack the capability of de novo purine synthesis^22^. The main purine source of the parasite is hypoxanthine, which is converted into inosine-5′-monophosphate (IMP) upon uptake, a precursor for all purine nucleotides. This reaction is catalyzed by hypoxanthine–guanine-xanthine-phosphoribosyl transferase (HGXPRT). A notable difference compared to the human host is the use of xanthine as a precursor for purine biosynthesis by HGXPRT. Although the parasites are capable of salvaging adenosine and converting it first into inosine and then into hypoxanthine through adenosine deaminase (ADA) and purine nucleoside phosphorylase (PNP), respectively, it has limited availability as erythrocytes possess adenosine kinases, turning it into AMP before uptake. *Plasmodium falciparum* mediates the uptake of purine derivatives from red blood cells via membrane transporters including the high-throughput parasitophorous vacuole membrane (PVM) channel and equilibrative nucleoside transporters (*Pf*ENT). Adenosine-5′-monophosphate (AMP) can only be obtained through IMP, as *Plasmodium falciparum* does not have nucleoside kinases. Guanosine-5′-monophosphate (GMP) can be produced through two pathways, one being through the aforementioned IMP transformed first into xanthosine-5′-monophosphate (XMP) by IMP dehydrogenase (IMPDH) and then in a consecutive step into GMP by the GMP synthase (GMPS). The other pathway is through the uptake of guanosine and guanine, substrates of PNP and HGXPRT, respectively. The pathway is discussed more extensively elsewhere^14,15,22,23^. Finally, the generated GMP and AMP is phosphorylated to the diphosphate form and then oxidized by the ribonucleotide reductase.

Despite the key importance of dNTP pools in maintaining the genomic integrity in the fast-proliferating *Plasmodium falciparum*, temporal changes in the nucleotide metabolism and the level of dNTPs have not been characterized in detail before. In this study, our aim was to analyze the dynamic changes in the dNTP pool during the intraerythrocytic life cycle and compare them to the expression pattern of the purine and pyrimidine metabolic enzymes using the high-quality RNA-Seq results available from databases. During our measurements, we also utilized WR99210, a drug that acts on the bifunctional TS-DHFR^24,25^, an enzyme of the de novo pyrimidine pathway and were able to characterize its impact on the dNTP pool of trophozoite stage parasites.

## Results

### Transcriptome analysis of the enzymes of the pyrimidine and purine biosynthetic pathways

As a first step, we aimed to in silico investigate the expression patterns of the enzymes involved in nucleotide biosynthesis in all intraerythrocytic stages. For the transcription analysis, we utilized high-quality transcriptomic data available on *plasmodb.org*, published by *Toenhake *et al*.*^26^.

Enzymes involved in de novo pyrimidine synthesis can be divided into two subsets in terms of their peak mRNA transcript level. Enzymes that catalyze reactions occurring earlier in the pathway have a slightly shifted peak at late trophozoite stage (Fig. 1B, around 30 h post-invasion (hpi)) compared to the enzymes involved further down the pathway, which peak at schizont stage (Fig. 1C, around 35 hpi). Furthermore, we observed the expression of the late subset of enzymes barely increases before the late ring stage, until around 20 h post-invasion. It raises the question of whether it makes pyrimidine nucleotides a limiting factor of replication and how it affects the genomic integrity of the parasite. Notably, all enzymes in both the pyrimidine and purine nucleotide pathway have a peak expression in late trophozoite to schizont stages, roughly at the end of the replicative cycle of the parasite and levels drop down in the last data points.

As carbamoyl-phosphate is the initial molecule from which synthesis starts a high demand for this molecule is expected. The mRNA level of CPSII—the enzyme responsible for the generation of carbamoyl-phosphate—is the most abundant from the early set of enzymes, thus supporting our aforementioned hypothesis. The subsequent enzymes DHOase, DHODH, OPRT, and ODC all follow the same trend of peak expression at 30 hpi, in the late trophozoite stage. Following the generation of UMP, the pyrimidine metabolic pathway branches out, as the sole pathway for cytosine derivative synthesis is through the turnover of UTP to CTP, while thymidine nucleotides originate from dUMP. Interestingly, the peak expression of CTP synthase can be observed approximately 5 h before any other enzymes involved in the de novo pyrimidine synthetic pathway despite the catalyzing enzymes of the precursor UMP having a peak expression later in the intraerythrocytic development.

Ribonucleotide reductase (RNR) converts the ribonucleotide diphosphates to the corresponding deoxyribonucleotide diphosphates, both in pyrimidine and purine pathways. RNR subunits R1 and R2 reach their peak transcriptome level at 35 hpi in the schizont stage. As RNR turns UDP into dUDP, it is phosphorylated into dUTP by a nucleotide diphosphate kinase (NDPK), which is then hydrolyzed by dUTPase to dUMP, the precursor of dTMP synthesis. dUTPase is an essential enzyme and it also has a peak expression in schizont stages, rather late in the developmental cycle^27^. Finally, one of the most characterized enzymes of the pathway is TS-DHFR, a common antimalarial drug target that is also included in the late subset of pyrimidine metabolic enzymes.

Based on the transcriptomic data, enzymes of the purine salvage pathway are expressed in a fairly similar manner (Fig. 2B). HGXPRT and PNP are involved in the salvage pathway and subsequent biosynthesis of both dGTP and dATP, therefore their high level of expression can potentially be explained by their high demand throughout the life cycle. The increased level of ADA expression might be due to the fact that adenosine is readily available from the bloodstream in higher concentrations^28^, and it can be used to generate both GMP and AMP. The peak expression of all enzymes coincides well with the early subset of pyrimidine metabolic enzymes highlighted prior at ca. 30 h post-invasion, corresponding to the late trophozoite stage. ADSS and ADSL enzymes are the least abundant amongst all purine nucleotide enzymes and their expression starts after 20 hpi, late in the ring stage. The expression profile shows a downward trend after 30 hpi in the later trophozoite and schizont stages, similar to the early subset of pyrimidine metabolic enzymes.

### Establishing the levels of all four canonical dNTPs in non-treated and WR99210 drug-treated parasites

After we analyzed the transcriptomic data generated by *Toenhake *et al.^26^ regarding the temporal dynamics of the expression profile for the nucleotide metabolic enzymes, we investigated the absolute and relative amount of the building blocks of DNA both in non-treated and treated, sorbitol-synchronized *P. falciparum* 3D7 cultures via a fluorescence-based deoxynucleotide-triphosphate (dNTP) incorporation assay^29^. For this, saponin-liberated parasites were centrifuged and washed with cold PBS two times to remove leftover debris. The lysate was stored overnight in methanol at − 20 °C to permeabilize the pellet. The isolation proceeded the next day with a heat shock, then isolates were centrifuged until clear supernatant was observed. Between the two consecutive centrifugation, the supernatant was transferred into clean centrifugation tubes. The methanol was evaporated in a vacuum concentrator until complete evaporation and the dNTPs then were resolved in RNase-free water. For all stages, three biological replicates were isolated and measured in three technical replicates (see the detailed isolation protocol in “Materials and methods” section). Isolated rings were aged approximately ~ 6 to 12 h, trophozoites ~ 30 to 36 h and schizont ~ 42 to 48 h post-invasion, respectively.

**Figure 3:**
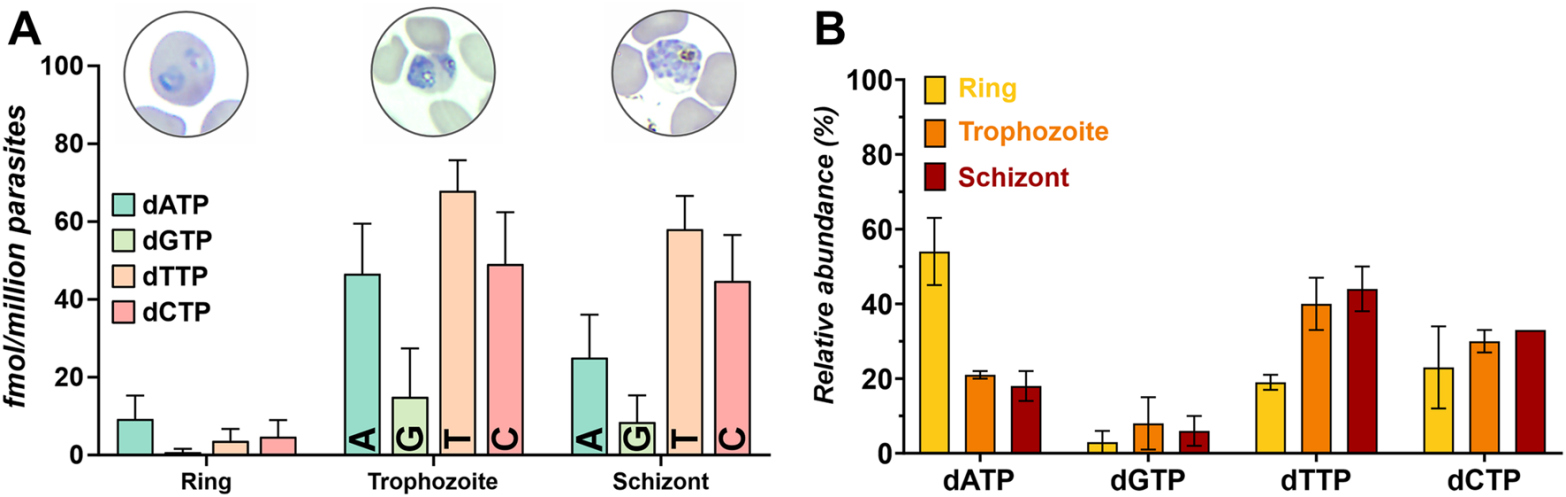
Deoxynucleotide triphosphate (dNTP) levels across the intraerythrocytic stages. (**A**) The absolute amount of dNTPs has been determined using a quantitative EvaGreen-based nucleotide incorporation assay. Representative microscopic images of ring (6–12 h post-infection), trophozoite (30–36 h post-infection) and schizont (42–48 h post-infection) stages are shown above their respective samples. Ring stages are still low in all nucleotides, with dATP (blue) present at the highest level. In the trophozoite stage almost all nucleotides increase significantly (p < 0.05) as compared to ring stage parasites and remain at similar levels during the schizont stage. In trophozoite and schizont stages, dTTP (orange) is the most abundant nucleotide and dCTP (pink) levels are elevated to the same amount as dATP. dGTP (green) is the least abundant dNTP across all stages. Results are shown in fmol per million parasites with error bars representing SEM values. (**B**) The relative abundance is expressed as the ratio of the amount of given nucleotide to the total dNTP amount of ring (yellow bars), trophozoite (orange bars) and schizont (red bars) stages, respectively. In the ring stage, dATP is the major dNTP species, which is surpassed by dTTP in the trophozoite and schizont stages. In all stages, dGTP has the lowest relative abundance. Results are presented in percentage of relative abundance with error bars representing SEM values.

The maintenance of the relative ratio of dNTPs during the proliferative life cycle of a cell is an essential and sensitive aspect of the accurate replication of DNA. The overall dNTP levels are low in ring stages (6–12 h post-invasion) with around 18 fmol total dNTP per million parasites (Fig. 3A). The most abundant dNTP is dATP at this stage, representing 54.4 ± 8.9% of the relative ratio of all dNTPs. dCTP and dTTP take up a comparable percentage with 23.1 ± 10.6% and 19.1 ± 1.9%, respectively. dGTP is the least abundant throughout the lifecycle, representing only 3.4 ± 2.9% at the ring stage (Fig. 3B). To note, dGTP is historically a difficult nucleotide to measure in *P. falciparum*, never having been measured before in such detail^22,30,31^_._

Based on the analysis of transcriptomic data, we expected the parasite to accumulate dNTPs by the trophozoite stage (30–36 h post-invasion), the main developmental cycle during which replication occurs. The absolute dNTP amount is indeed substantially elevated compared to ring stage parasites, with a tenfold increase to around 180 fmol total dNTP per million parasites (Fig. 3A). The relative ratio of dNTPs in the trophozoite stage is as follows: dTTP is the most abundant with 40.1 ± 6.5%, then dATP and dCTP represent a similar ratio of 21.4 ± 1.3% and 30.1 ± 3.2%, respectively, while dGTP is the lowest amongst all nucleotides with only 8.4 ± 7.4% (Fig. 3B). Note that the relative levels have undergone a major shift compared to the ring stage, which is potentially due to the changes previously observed in the expression profile of genes involved in the nucleotide metabolism for both purine and most notably, the *de novo* pyrimidine biosynthesis.

Approaching the final intraerythrocytic stage, the schizont stage (42–48 h post-invasion), both the absolute level (~ 140 fmol total dNTP per million parasites) and the relative ratio of all dNTPs preserve quite similar proportions as seen in trophozoites (Fig. 3A,B): dTTP is still the most abundant with 43.5 ± 6.2%, dATP and dCTP accounting for 17.8 ± 3.8% and 32.8 ± 0.4%, respectively. As seen in all stages prior, dGTP is the least abundant with 6.0 ± 3.7%.

**Figure 4:**
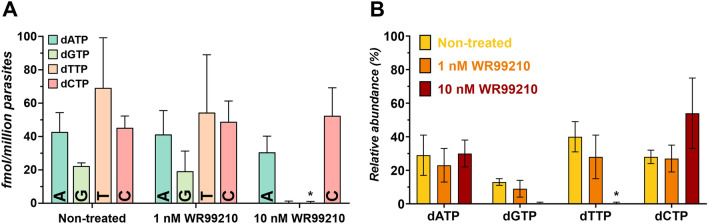
Impact of WR99210 treatment on the dNTP pool of trophozoite stage parasites. (**A**) The dNTP pool of trophozoite stage (30–36 h post-invasion) parasites has been characterized following a 24-h drug exposure to WR99210. The samples are isolated from trophozoites of the same culture, splitting them before drug addition. The drug WR99210 has not shown any significant impact on the dNTP levels at 1 nM concentration, as indicated in the bar graph. However, we have found that at the 10 nM WR99210 concentration dTTP (orange bar) and dGTP (green bar) levels are diminished, but only dTTP has been found to decrease statistically significantly (* corresponds to p < 0.001), while dGTP still dropped below the limit of detection. Results are expressed in fmol per million parasites, with error bars indicating SEM values. (**B**) The relative ratio of the dNTPs in each drug-treated sample are expressed in relative percentage to the total dNTP amount. Non-treated samples (yellow bars) and 1 nM WR99210 treated samples (orange bars) show no significant changes. The 10 nM WR99210 treated (red bar) samples have diminished ratio of dTTP and dGTP, which consequentially increases the overall ratio of dATP and dCTP. Error bars indicate SEM values.

We wanted to further analyze the dynamic changes in the dNTP following drug exposure. To test it, we have chosen a pyrimidine metabolic inhibitor, WR99210 that has a subnanomolar antimalarial effect and is generally used for the selection of *Plasmodium* transfectants. WR99210 acts on the bifunctional parasitic enzyme thymidylate synthase-dihydrofolate reductase (TS-DHFR)^32^. For the treatment, the highest drug concentration was chosen based on a previous study that characterized transcriptional changes under 10 nM WR99210 treatment^32^. Furthermore, we wished to analyze a lower, 1 nM WR99210 concentration to see how a concentration closer to the EC_50_ (~ 0.1 nM) impacts the dNTP pool^24,25^. Parasites were exposed to the drug for 24 h, from ring stage parasites (aged ~ 6 to 12 h) throughout the trophozoite stage and isolated as late-trophozoites (aged ~ 30 to 36 h), thus covering the time frame of replication completely. Interestingly, parasites seem to be able to survive the effect of the drug at 1 nM drug concentration. Compared to the non-treated culture, we see no significant change neither in overall dNTP levels (Fig. 4A) nor in the relative ratio (Fig. 4B). However, at the 10 nM WR99210 concentration, the absolute amount of dTTP is diminished compared to the non-treated isolates (Fig. 4A), while dATP and dCTP do not change significantly. Surprisingly, in addition to diminished dTTP levels, dGTP has also reduced under the detection limit despite being synthesized in the purine pathway.

Normally, before the replication begins, cells accumulate nutrients, including nucleotides to prepare for the DNA replication and the development of daughter cells^33^. We found that in *Plasmodium falciparum* the youngest intraerythrocytic stage, the ring stage contains only low levels of dNTP across all nucleotides. By the time parasites reach the trophozoite stage when replication occurs^33^, they already accumulate a significant amount of dNTP within the short, 24-h time window between the two stages. We found that all nucleotides increased significantly from ring to trophozoite stage (Welch-test, p < 0.05, n ≥ 3), except dGTP, which had a high variance to establish a statistically significant difference, albeit the quantification method clearly indicates an increased amount. The observed ratio stays constant until the very end of the life cycle, when schizonts develop into the daughter cell merozoites. Additionally, treating parasites with WR99210 significantly reduces the dTTP and also dGTP levels at 10 nM concentration in late trophozoites (aged ~ 30 to 36 h), but not in 1 nM WR99210 treated parasites.

## Discussion

A balanced dNTP pool is indispensable for DNA synthesis as disturbances to it can cause mutations. *Plasmodium falciparum* parasites start DNA replication in the early trophozoite stage, approximately 24–26 h post-invasion. Before this, young parasitic cells corresponding to the ring and early trophozoite stage are in G1 phase of the cell cycle with a single haploid nucleus preparing for the intense replication that follows^33^. DNA replication is associated with the S phase in standard eukaryotic cells, but malaria parasites deviate from this classical model as each parasite undergoes multiple replications asynchronously before mitosis^34^ and produces on average 16–20 new daughter cells^33^.

During the in silico transcriptional analysis, we found that only a modest synthesis of the nucleotide building blocks of DNA starts early in the ring stage. Although the expression of enzymes involved in the purine salvage pathway begins already in ring stage, only low levels of dATP and very low dGTP can be found at this stage. In ring stage parasites dATP is the most abundant of all dNTPs, but the overall dNTP level is low. Pyrimidine nucleotides dTTP and dCTP show a similar level compared to each other but are still at a fairly lower level than dATP, indicating that possibly the de novo pathway is still slow in the biosynthetic process. Host erythrocytes are abundant in purine bases and nucleosides and parasites salvage these through membrane transporters^22^. This might be the reason behind dATP being the most abundant at the first stage. However, the synthesis of deoxynucleotides requires ribonucleotide reductase, and expression of this enzyme can only be observed from the late ring stage (20 hpi) as parasite cells reach the end of the G1 phase. A possible explanation for this phenomenon could be that a subset of enzymes or dNTPs are inherited by daughter cells from the mother cell. Later stages in the intraerythrocytic life cycle present a reduced relative ratio of dATP compared to the ring stage and stays around 20% throughout the rest of the intraerythrocytic life cycle.

In all stages, dGTP is the least abundant of all nucleotides. This was observed in other eukaryotic and prokaryotic cells as well and proposed that as a control measure, dGTP is kept at low levels^35–37^. It was suggested that opposite to T sites in the DNA strand, dGTP as the incorrect and dATP as the correct nucleotide compete to be incorporated. Misincorporation of dGTP poses a considerable amount of stress on the mismatch repair (MMR) system and elevated levels of dGTP might lead to error catastrophe^35^.

The expression profile of the early subset of de novo pyrimidine pathway enzymes start to elevate during ring stage and reach their peak expression during late trophozoite stage. This coincides well with the observation that dTTP reaches the highest relative ratio in trophozoite stage and maintains a high relative ratio throughout schizont stage. However, the late subset of de novo pyrimidine biosynthesis enzymes reach their peak expression level during early schizont stage. After dTTP, the relative ratio of dCTP is the highest both in trophozoite and schizont stages. Surprisingly, dCTP levels are comparable to dATP levels, an observation that is not yet understood considering the low GC content of the *Plasmodium falciparum* genome.

An important aspect of our study was to characterize the impact of drug treatment on the dNTP pool of *Plasmodium falciparum* parasites. Here, we used WR99210, a drug acting on thymidylate synthase-dihydrofolate reductase of the parasite to investigate the changes in the nucleotide levels. Interestingly, we have found that it decreases dTTP levels at 10 nM concentration, but not at 1 nM, despite the subnanomolar EC50 value of the drug. Surprisingly, at 10 nM drug concentration not only the dTTP, but the dGTP level has also decreased under detection limit in late-trophozoite (aged 30–36 h post-invasion) isolates, despite dGTP being a purine nucleotide that is synthesized in a completely different biosynthetic pathway. The reason behind this might be an interesting link between the purine and pyrimidine biosynthesis via the thymidylate kinase enzyme that can recognize both nucleotide monophosphates as substrate. However, WR99210 has not been previously suggested to effect TK activity as it is a folate analogue and acts on the DHFR part of the TS-DHFR enzyme^38^. Therefore, we conclude that a more indirect cause may also be involved in this phenomenon. Further studies out of the scope of this present work are needed to provide mechanistic insights to account for this result.

In this work, our primary aim was to give a detailed overview of dNTP levels and the expression profile of the nucleotide metabolic enzymes in *Plasmodium falciparum* in a stage-specific manner. We were able to characterize the dNTP pool and its relative ratio distribution in ring, trophozoite and schizont stage parasites alike. Remarkably, we were able to measure dGTP in every intraerythrocytic stage and could show that it is indeed the least abundant dNTP in all stages. To demonstrate that the method is capable of detecting dynamic changes in dNTP pool, we utilized WR99210 treatment on parasites and measured its effect on deoxynucleotide levels. The ability to detect dNTP level changes might contribute to understanding and characterizing the mode of action of antimalarials and developing new drugs targeting the purine and pyrimidine metabolic pathways.

## Materials and methods

### Parasite culture and maintenance

*Plasmodium falciparum* 3D7 strain was grown in A + erythrocytes (Hungarian National Blood Transfusion Services, Budapest, Hungary) in complete malaria culture medium of RPMI 1640 w/l-glutamine, HEPES, and NaHCO_3_ (VWR Chemicals, Radnor, PA, USA) supplemented with 37 µM hypoxanthine (VWR Chemicals, Radnor, PA, USA), 1.25 g/L Albumax I and 50 mg/L gentamycin (Gibco from Thermo Fisher Scientific, Waltham, MA, USA). Cultures were maintained at 3% hematocrit, 37 °C in an atmosphere of 5% O_2_, 5% CO_2_, and 90% N_2_. Cultures were synchronized regularly by the sorbitol-synchronization method. During this method cultures with at least 2% parasitemia containing mainly rings were incubated with 5% sorbitol (Sigma-Aldrich, Merck Group, Darmstadt, Germany) for 10 min at 37 °C after centrifugation. After washing, parasites were further cultured as described above.

### dNTP isolation

Ring-stage cultures with at least 5% parasitemia were sorbitol-synchronized (two consecutive synchronizations, 6 h apart). The rest of the culture was maintained further until the upcoming isolation procedures for the different stages as checked by light microscopy smears. For each stage sample, 300 µl pellets were harvested. Isolated ring parasites were ~ 6 to 12 h, trophozoites ~ 30 to 36 h and schizonts ~ 42 to 48 h old at harvest. In case of the WR99210 treated parasites, the drug was added to the parasites following the second sorbitol synchronization at ring stage and incubated until the late-trophozoite stage (~ 30 to 36 h old). We diluted the drugs using complete malaria culture medium for both concentrations. The collected parasitized pellet was resuspended in cold phosphate buffer saline (PBS), then 0.02% saponin containing PBS of the same volume (amount of PBS 1 × and 0.02% saponin containing PBS 1 × should be added as final hematocrit should be around 5%) added. Shaking by reversing the tube and incubation at room temperature for 5 min followed. The solution was centrifuged at 1000*g* and washed with cold PBS two times. The lysate was stored overnight in 500 µL 60% methanol at − 20 °C. The isolation proceeded the next day, starting with a 5 min 95 °C heat shock, then isolates were centrifuged at 11,000*g* for 20 min twice. Between the two consecutive centrifugation, the supernatant was transferred into clean centrifugation tubes. The methanol was evaporated in a vacuum concentrator (Eppendorf Vacufuge Concentrator System) at 45 °C until complete evaporation. The dNTPs then were resolved in 100 µl RNase free water. For all stages three biological replicates were isolated and all biological samples were measured in three technical replicates. Smear samples were collected before each isolation to verify the parasitic life stage and calculate parasitemia. Thin blood smears were stained with Kwik-Diff Stain Kit (Thermo Fisher Scientific, Waltham, MA, USA) according to product instructions and were examined by light microscopy.

### Fluorescence-based dNTP quantification method

dNTP containing samples were measured by a novel fluorescence-based method developed by *Purhonen *et al.^29^. We used a long synthetic oligonucleotide (197-nt) as a template that contains a primer-binding site directly adjacent to the sole dNTP-detection site of the nucleotide to be determined in the given experiment, which is followed by a long stretch of other bases, as a signal amplification sequence. The principle of the assay is that for each dNTP to be determined, the 197-nt template contains only one single corresponding nucleotide (e.g. for determination of dGTP, there is one single cytosine in the template). In addition, for each dNTP to be determined, the polymerization reaction master mix contains only the other three dNTPs, hence the samples provide the sole source of the dNTP to be determined. During polymerization, the double-stranded DNA product is formed and binds the EvaGreen dye. Binding of the EvaGreen dye to the double-stranded DNA elicits high fluorescence. The length of the template allows for appropriate signal amplification. For the PCR reaction, Q5^®^ High-Fidelity DNA polymerase (New England Biolabs) was used. The 2X master mix for the qPCR measurements was the following: 0.275 µM primer (Merck KGaA, Darmstadt, Germany), 0.25 µM template (IDT, Coralville, Iowa, USA), 50 µM dNTP mix (always excluding the dNTP to be measured in the given experiment such as to limit its source to the sample), and 1.25 µM EvaGreen (Biotum). 20 U/ml Q5^®^ High-Fidelity DNA polymerase was added for dATP and dTTP, 10 U/ml for dGTP and dCTP measurement. The 96-well plates (Applied Biosystems MicroAmp^®^ Optical plate) contained 10 µl final reaction volume (5 µl sample, 5 µl 2X master mix). The samples contained the isolated dNTPs from the desired stage as described in the dNTP isolation section. For all examined stages, we measured three biological replicates in three technical replicates. The qPCR program was performed on a Bio-Rad CFX96 qPCR instrument according to the step-by-step protocol in the Supplementary file of the *Purhonen *et al*.* article^29^. Briefly, the qPCR program was as follows: first, 10 s at 95 °C, then 1 s at 75 °C to determine a baseline fluorescence reading. The cycle starts with a 1 s read-out of the fluorescence at 66 °C, which we use to monitor the progress of the qPCR reaction. The read-out is followed by a 5 min annelation–elongation step at 66 °C. The cycle should be repeated 10 times for dATP, 7 times for dTTP and dCTP and 3 times for dGTP. After the suitable number of cycles, a 5 s read-out determines the end-point fluorescence of the qPCR reaction at 75 °C. The reason the read-out occurs at 75 °C is that it gives a better signal-to-noise ratio and more reliable data. The data has been analyzed in BioRad CFX Maestro Software and analyzed using GraphPad Prism.

### Analysis of transcriptomic data

Transcriptome analysis has been carried out using the PlasmoDB database (*plasmodb.org*), where transcriptomic FPKM values have been manually collected, organized, and plotted for all purine and pyrimidine biosynthetic enzymes from the RNA-Seq dataset generated by *Toenhake* et al.^26^. We summarized the accession ID and name of all genes investigated as a part of this study in Table 1.

**Table 1:** Gene ID of all the investigated nucleotide metabolic enzymes.

Gene ID on PlasmoDB	Name
PF3D7_1029600	Adenosine deaminase (ADA)
PF3D7_0513300	Purine nucleoside phosphorylase (PNP)
PF3D7_1012400	Hypoxanthine–guanine–xanthine phosphoribosyl transferase (HGXPRT)
PF3D7_0920800	Inosine-5′-monophosphate dehydrogenase (IMPDH)
PF3D7_1012600	Guanosine-5′-monophosphate dehydrogenase (GMPS)
PF3D7_1354500	Adenylosuccinate synthetase (ADSS)
PF3D7_0206700	Adenylosuccinate lyase (ADSL)
PF3D7_1329400	Adenosine-5′-monophosphate deaminase (AMPD)
PF3D7_1308200	Carbamoyl-phosphate synthetase II (CPSII)
PF3D7_1344800	Aspartate transcarbamoylase (ATCase)
PF3D7_1472900	Dihydroorotase (DHOase)
PF3D7_0603300	Dihydroorotate dehydrogenase (DHODH)
PF3D7_0512700	Orotate phosphoribosyl transferase (OPRT)
PF3D7_1023200	Orotidine-5′-monophosphate decarboxylase (ODC)
PF3D7_1366500	Nucleoside diphosphate kinase (NDPK)
PF3D7_1410200	Cytidine-5′-triphosphate synthase (CTPS)
PF3D7_1437200	Ribonucleotide reductase large subunit (RNR R1)
PF3D7_1405600	Ribonucleotide reductase small subunit (RNR R1)
PF3D7_0417200	Thymidylate synthase-dihydrofolate reductase (TS-DHFR)
PF3D7_1127100	Deoxyuridine-5′-triphosphate nucleotide hydrolase (dUTPase)
PF3D7_1251300	Thymidylate kinase (TK)

## Data Availability

The dataset analyzed during the current study is available in the PlasmoDB repository, https://plasmodb.org/plasmo/app/record/dataset/DS_ee861a9187.
