# Author Correction: Characterization of the dynamics of *Plasmodium falciparum* deoxynucleotide-triphosphate pool in a stage-specific manner

**DOI:** 10.1038/s41598-022-27314-4

**Published:** 2023-01-03

**Authors:** Réka Babai, Richard Izrael, Beáta G. Vértessy

**Affiliations:** 1 Malaria Research Laboratory, Institute of Enzymology, Research Centre for Natural Sciences, Budapest, 1117 Hungary; 2 George A. Olah Doctoral School of Chemistry and Chemical Technology, BME Budapest University of Technology and Economics, Budapest, 1111 Hungary; 3 Department of Applied Biotechnology and Food Sciences, BME Budapest University of Technology and Economics, Budapest, 1111 Hungary; 4 Doctoral School of Multidisciplinary Medical Sciences, University of Szeged, Szeged, 6720 Hungary

Correction to: *Scientific Reports* 10.1038/s41598-022-23807-4, published online 19 November 2022

The original version of this Article contained errors in the Introduction, the Discussion, and the legend of Figure 1.

Firstly, in the Introduction,

“Finally, thymidine kinase (TK) phosphorylates dTMP to provide the properly phosphorylated dTTP for replication.”

now reads:

“Finally, thymidylate kinase (TK) and nucleotide diphosphate kinase (NDPK) phosphorylates dTMP to dTDP and dTTP, respectively, to provide the properly phosphorylated form for replication.”

Secondly, in the Discussion,

“The reason behind this might be an interesting link between the purine and pyrimidine biosynthesis via the thymidine kinase enzyme that can recognizes both nucleotide monophosphates as substrate.”

now reads:

“The reason behind this might be an interesting link between the purine and pyrimidine biosynthesis via the thymidylate kinase enzyme that can recognize both nucleotide monophosphates as substrate.”

Finally, in the legend of Figure 1,

“Finally, dUMP is turned into thymidine-5′-phosphate (dTMP) by the bifunctional thymidylate synthase-dihydrofolate reductase (TS-DHFR), that is then phosphorylated to dTTP by thymidine kinase (TK).”

now reads:

“Finally, dUMP is turned into thymidine-5′-phosphate (dTMP) by the bifunctional thymidylate synthase-dihydrofolate reductase (TS-DHFR), that is then turned into dTTP in consecutive phosphorylation steps by thymidylate kinase (TK) and nucleotide diphosphate kinase (NDPK).”

The original Article has been corrected.

